# Barcoding Fauna Bavarica: 78% of the Neuropterida Fauna Barcoded!

**DOI:** 10.1371/journal.pone.0109719

**Published:** 2014-10-06

**Authors:** Jérome Morinière, Lars Hendrich, Axel Hausmann, Paul Hebert, Gerhard Haszprunar, Axel Gruppe

**Affiliations:** 1 Bavarian State Collection of Zoology (SNSB - ZSM), München, Germany; 2 Biodiversity Institute of Ontario (BIO), University of Guelph, Guelph, Canada; 3 Institute of Animal Ecology (TU München), Freising, Germany; Onderstepoort Veterinary Institute, South Africa

## Abstract

This publication provides the first comprehensive DNA barcode data set for the Neuropterida of Central Europe, including 80 of the 102 species (78%) recorded from Bavaria (Germany) and three other species from nearby regions (Austria, France and the UK). Although the 286 specimens analyzed had a heterogeneous conservation history (60% dried; 30% in 80% EtOH; 10% fresh specimens in 95% EtOH), 237 (83%) generated a DNA barcode. Eleven species (13%) shared a BIN, but three of these taxa could be discriminated through barcodes. Four pairs of closely allied species shared barcodes including *Chrysoperla pallida* Henry et al., 2002 and *C. lucasina Lacroix,* 1912*; Wesmaelius concinnus* (Stephens, 1836) and *W. quadrifasciatus* (Reuter, 1894); *Hemerobius handschini* Tjeder, 1957 and *H. nitidulus* Fabricius, 1777*;* and *H. atrifrons* McLachlan, 1868 and *H. contumax* Tjeder, 1932. Further studies are needed to test the possible synonymy of these species pairs or to determine if other genetic markers permit their discrimination. Our data highlight five cases of potential cryptic diversity within Bavarian Neuropterida: *Nineta flava* (Scopoli, 1763), *Sympherobius pygmaeus* (Rambur, 1842), *Sisyra nigra* (Retzius, 1783), *Semidalis aleyrodiformis* (Stephens, 1836) and *Coniopteryx pygmaea* Enderlein, 1906 are each split into two or three BINs. The present DNA barcode library not only allows the identification of adult and larval stages, but also provides valuable information for alpha-taxonomy, and for ecological and evolutionary research.

## Introduction

**Figure 1:**
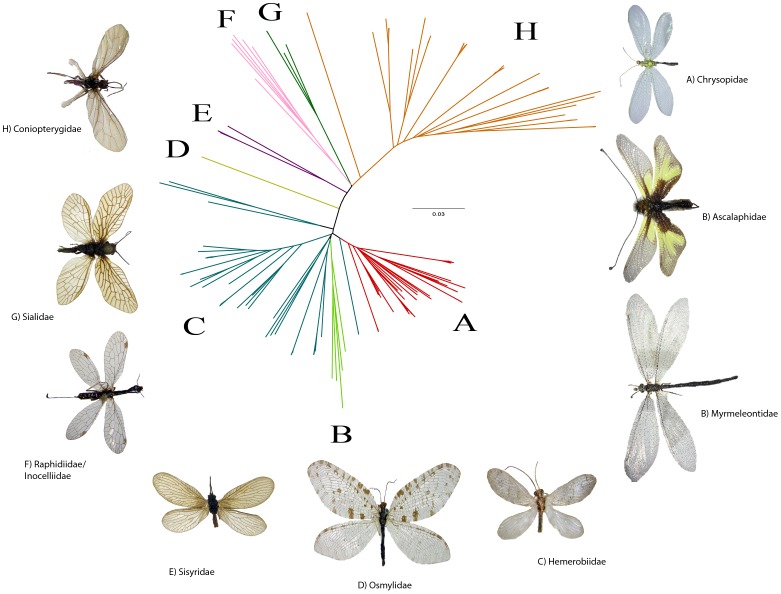
Neighbour joining tree (established in BOLD – Radial tree layout was performed in Figtree).

The comparatively small clade of holometabolous Neuropterida contains three insect orders (Rhaphidioptera, Megaloptera, Neuroptera) with about 6.300 described species worldwide [1]. Part of the superorder Endopterygota and closely related to beetles (Coleoptera), they are usually considered an unranked taxon [2]. Well-known members of Neuropterida are the snakeflies, dobsonflies, fishflies, lacewings and antlions. Some neuropterans are economically important, as the larvae of Chrysopidae and Hemerobiidae are used for the biocontrol of pest species on agricultural crops [3], [4]. Saure [5], [6], [7] reported 115 species for Germany and 97 species for Bavaria, but more recent studies have raised the count for Bavaria to 102 species [8]. This study provides COI barcode sequences for 80 of these species, including representatives of all 35 known genera (**Fig. 1** **and Fig. S1**).

In 2006 the Bavarian State Collection of Zoology (ZSM) started a close collaboration with the Biodiversity Institute of Ontario (‘BIO’, Guelph, Canada) to assemble a DNA barcode library for all animals, plants and fungi known to occur in Bavaria in the framework of the International Barcode of Life Initiative (‘iBOL’). Over the past seven years, the ZSM submitted tissue samples from more than 150,000 identified vouchers belonging to more than 40,000 insect species. Sequencing was performed at the Canadian Centre for DNA Barcoding (‘CCDB’, Guelph, Canada). Photographs and geo-referenced label data, barcode sequences and trace files for all vouchers are available on the BOLD database [9], [10]. The present DNA barcode data set was produced as part of the Barcoding Fauna Bavarica (BFB) campaign which is a 10 year project (2009–2018) of the ZSM. The goal of this project is to create a DNA barcode library for all Bavarian species. Bavaria represents the largest of all German states with a landmass of 70,000 km^2^. It also harbors the highest biodiversity of all German states (with high altitude biomes, foothill areas and forested lowlands), with at least 35,000 animal species reported [11], representing a significant portion of the Central European fauna.

## Material and Methods

### Ethics statements

Field work permits were issued by the responsible state environmental office of Bavaria [Bayerisches Staatsministerium für Umwelt und Gesundheit, Munich, Germany, project: “Barcoding Fauna Bavarica”, reference number 62e-U8645.8-2008/3-17]. The study sites comprise state forests, public land and protected areas. We confirm that the field studies did not involve any protected species by European or national laws.

### Specimens

We selected 286 specimens of Neuropterida from the collection of A.G. (which will later be deposited in the ZSM) as well as from material in the ZSM (**Table 1**). Additional specimens of Neuropterida collected by Malaise traps in the Bavarian Forest National Park were also included. For 20 species known from Bavaria we used vouchers from outside Bavaria, including three species (*Pseudomallada inornata* (Navás, 1901), *Coniopteryx hoelzeli Aspöck, 1964*, *C. drammonti* Rousset, 1964), which are likely to occur in Bavaria, but have not yet been recorded. Specimens were determined to a species level according to Aspöck et al. [12] following the nomenclature of Aspöck et al. [2]. Photos of all specimens and all sequence records are available on BOLD (public data set DS-NEUBFB; dx.doi.org/10.5883/DS-NEUBFB), while sequence data are also on GenBank (cf. accession numbers in Appendix S1).

**Table 1:** Species list including species (BIN) origin, number of specimen (n), as well as Mean and Max intraspecific differences (ISD).

Family	Species	Country	BIN	n	Mean ISD	Max ISD	Nearest Species (NS)	Distance to NS
**Ascalaphidae**	***Libelloides coccajus*** (Denis & Schiffermüller, 1775)	FR	ACD2658	1	–	–	*Myrmelon bore*	14.75
**Chrysopidae**	***Chrysopa dorsalis*** Burmeister, 1839	DE	ABV5183	2	0.46	0.46	*Chrysopa walkeri*	5.86
	***Chrysopa formosa*** Brauer, 1851	IT	ACF7085	1	–	–	*Chrysopa walkeri*	5.53
	***Chrysopa pallens*** (Rambur, 1838)	DE	AAZ4625	1	–	–	*Chrysopa walkeri*	8.36
	***Chrysopa perla*** (Linnaeus, 1758)	DE	AAJ5114	6	0.18	0.3	*Chrysopa walkeri*	7.87
	***Chrysopa phyllochroma*** Wesmael, 1841	DE	ABU9803	1	.	–	*Chrysopa walkeri*	7.67
	***Chrysopa viridana*** Schneider, 1845	FR	ACF7175	1	.	–	*Pseudomallada flavifrons*	6.34
	***Chrysopa walkeri*** McLachlan, 1893	FR	ACF7899	1	.	–	*Chrysopa formosa*	5.53
	***Chrysoperla carnea*** Stephens, 1836	DE, FR	AAB0373	6	0.09	0.3	*Chrysoperla lucanisa*	0.76
	***Chrysoperla lucasina*** Lacroix, 1912	DE	AAB0373	6	0.42	0.92	*Chrysoperla pallida*	0
	***Chrysoperla pallida*** Henry et al., 2002	DE	AAB0373	4	0.08	0.15	*Chrysoperla lucanisa*	0
	***Chrysotropia ciliata*** (Wesmael, 1841)	DE	AAJ3493	7	0.3	0.61	*Nineta inpunctata*	10.59
	***Cunctochrysa albolineata*** (Killington, 1935***)***	FR	ABW9035	1	.	–	*Hypochrysa elegans*	7.84
	***Hypochrysa elegans*** (Burmeister, 1839)	DE	ACF9606	2	0	0	*Pseudomallada prasinus*	7.84
	***Nineta flava*** (Scopoli, 1763)	DE	ABW7306	2	0.3	0.3	*Cunctochrysa albolineata*	2.95
	***Nineta inpunctata*** (Reuter, 1894)	DE	ABW9495	1	–	–	*Nineta vittata*	4.23
	***Nineta pallida*** (Schneider, 1846***)***	DE	ACF6511	1	–	–	*Nineta flava*	6.84
	***Nineta vittata*** (Wesmael, 1841)	DE	ABW7143	1	–	–	*Nineta inpunctata*	2.95
	***Nothochrysa capitata*** (Fabricius, 1793)	DE	ABW9405	2	–	–	*Nineta flava*	8.87
	***Nothochrysa fulviceps*** (Stephens, 1836)	AT	ACF9634	1	–	–	*Pseudomallada flavifrons*	7.51
	***Peyerimhoffina gracilis*** (Schneider, 1851)	DE	AAY1798	2	0.3	0.3	*Chrysopa viridana*	8.86
	***Pseudomallada abdominalis*** (Brauer, 1856)	AT, FR	ACF8793	2	1.7	1.7	*Pseudomallada prasinus*	2.79
	***Pseudomallada flavifrons*** (Brauer, 1851)	DE	AAL0885	1	–	–	*Pseudomallada inornata*	5.69
	***Pseudomallada inornata*** (Navás, 1901)	DE	ACG0517	1	–	–	*Pseudomallada flavifrons*	5.69
	***Pseudomallada prasinus*** (Burmeister, 1839)	DE	ACF9046	2	0	0	*Pseudomallada ventralis*	1.85
	***Pseudomallada ventralis*** (Curtis, 1834)	DE	ABU9179	2	0	0	*Pseudomallada ventralis*	1.85
**Coniopterygidae**	***Aleuropteryx loewii*** Klapálek, 1894	DE	ACG4956	3	0	0	*Semidalis aleyrodiformis*	12.54
	***Coniopteryx aspoecki*** Kis, 1967	DE	AAV8086	2	0.15	0.15	*Coniopteryx borealis*	15.41
	***Coniopteryx borealis*** Tjeder, 1930	DE, FR	AAV8088	14	0.45	1.38	*Coniopteryx tineiformis*	14.49
	***Coniopteryx drammonti*** Rousset, 1964	DE	ACJ8029	1	–	–	*Coniopteryx haematica*	12.12
	***Coniopteryx esbenpeterseni*** Tjeder, 1930	DE, FR	AAU4144	7	0.61	1.23	*Coniopteryx lentiae*	1.16
	***Coniopteryx haematica*** McLachlan, 1868	DE	ACG0278	3	0.1	0.16	*Coniopteryx drammonti*	12.12
	***Coniopteryx hoelzeli*** Aspöck, 1964	DE	ACJ9063	1	.	–	*Coniopteryx haematica*	16.28
	***Coniopteryx lentiae*** Asp**öck** & Asp**ö**ck, 1964	DE	AAU4144	4	0.55	0.66	*Coniopteryx esbenpeterseni*	1.16
	***Coniopteryx pygmaea*** Enderlein, 1906	DEDEDE	AAV8087AAU4143ACJ9303	441	9.08	14.83	*Coniopteryx haematica*	14.44
	***Coniopteryx tineiformis*** Curtis, 1834	DE, FR	AAU2590	18	0.65	1.23	*Coniopteryx borealis*	14.49
	***Conwentzia pineticola*** Enderlein, 1905	DE	AAU1711	5	0.52	0.92	*Conwentzia psociformis*	11.4
	***Conwentzia*** ** ***psociformis*** (Curtis, 1834)	DEDE	ACF6246ACJ9308	22	9.13	13.48	*Conwentzia pineticola*	11.4
	***Helicoconis lutea*** (Wallengren, 1871)		AAV6876	2	0	0	*Coniopteryx pygmaea*	22.4
	***Semidalis aleyrodiformis*** (Stephens, 1836)	DEDE	AAU2412AAU2413	43	2.2	3.77	*Conwentzia pineticola*	15.14
**Hemerobiidae**	***Drepanepteryx phalaenoides*** (Linnaeus, 1758)	DE, FR	AAL1720	9	0.27	0.61	*Wesmaelius subnebulosus*	13.73
	***Hemerobius atrifrons*** McLachlan, 1868	DE	ACF6575	1	–	.	*Hemerobius contumax*	0
	***Hemerobius contumax*** Tjeder, 1932	AT	ACF6575	1	–	.	*Hemerobius atrifrons*	0
	***Hemerobius fenestratus*** Tjeder, 1932	DE	AAU3559	4	0	0	*Hemerobius pini*	7.58
	***Hemerobius handschini*** Tjeder, 1957	AT	ABU9615	2	0.3	0.3	*Hemerobius nitidulus*	0.15
	***Hemerobius humulinus*** Linnaeus, 1758	DE, FR	AAG0892	10	0	0	*Hemerobius stigma*	5.2
	***Hemerobius lutescens*** Fabricius, 1793	DE, UK	AAU3560	3	0	0	*Hemerobius micans*	7.85
	***Hemerobius marginatus*** Stephens, 1833	DE, UK	AAP2910	4	0.92	1.7	*Hemerobius humulinus*	9.25
	***Hemerobius micans*** Olivier, 1792	DE, FR	AAU2797	8	0.57	1.08	*Hemerobius humulinus*	5.24
	***Hemerobius nitidulus*** Fabricius, 1777	DE	ABU9615	3	0.1	0.15	*Hemerobius handschini*	0.15
	***Hemerobius pini*** Stephens, 1836	DE	ABZ6750	5	0.24	0.3	*Hemerobius atrifrons*	4.08
	***Hemerobius stigma*** Stephens, 1836	DE	ABZ6748	3	0.1	0.15	*Hemerobius humulinus*	5.2
	***Megalomus hirtus*** (Linnaeus, 1761)	DE	ABU9398	2	0.3	0.3	*Hemerobius lutescens*	12.91
	***Micromus lanosus*** (Zeleny, 1962)	FR	ACF8233	1	–	–	*Micromus paganus*	11
	***Micromus paganus*** (Linnaeus, 1767)	DE, FR	ABU9392	3	0.1	0.15	*Micromus lanosus*	11
	***Micromus variegatus*** (Fabricius, 1793)	DE, FR	AAP8424	4	0	0	*Wesmaelius malladai*	13.64
	***Psectra diptera*** (Burmeister, 1839)	DE	ABU9130	1	–	–	*Hemerobius humulinus*	13.99
	***Sympherobius elegans*** (Stephens, 1836)	DE	ACF6278	1	–	–	*Sympherobius pellucidus*	12.19
	***Sympherobius fuscescens*** (Wallengren, 1863)	DE	ABU9201	1	–	–	*Sympherobius pellucidus*	12.57
	***Sympherobius klapaleki*** Zeleny, 1963	DE	ACG0423	1	–	–	*Sympherobius pellucidus*	10.43
	***Sympherobius pellucidus (***Walker, 1853)	AT, DE	ACF7486	2	0.15	0.15	*Sympherobius klapaleki*	10.43
	***Sympherobius pygmaeus*** (Rambur, 1842)	DEDE	ACF9381ACG0292	21	2.59	3.12	*Sympherobius elegans*	12.4
	***Wesmaelius concinnus*** (Stephens, 1836)	DE	ABU9030	2	0	0	*Wesmaelius quadrifasciatus*	0
	***Wesmaelius malladai*** (Navás, 1925)	AT, FR	ABV4412	2	0.15	0.15	*Wesmaelius subnebulosus*	5.74
	***Wesmaelius nervosus*** (Fabricius, 1793)	DE, FR	ACF3795	2	0	0	*Wesmaelius subnebulosus*	5.12
	***Wesmaelius quadrifasciatus*** (Reuter, 1894)	AT	ABU9030	1	.	–	*Wesmaelius concinnus*	0
	***Wesmaelius subnebulosus*** (Stephens, 1836)	DE	No BIN available	1	.	–	*Wesmaelius nervosus*	5.12
**Inocellidae**	***Inocellia crassicornis*** (Schummel, 1832)	DE	ACF8844	1	–	–	*Phaeostigma notata*	16.42
**Mrymeleontidae**	***Distoleon tetragrammicus*** (Fabricius, 1798)	DE	ACD5335	1	–	–	*Euroleon nostras*	15.13
	***Euroleon nostras*** (Geoffroy in Fourcroy, 1785)	DE	AAV7116	2	0	0	*Myrmeleon bore*	13.49
	***Myrmeleon bore*** (Tjeder, 1941)	DE	AAH2239	1	–	–	*Euroleon nostras*	13.49
	***Myrmeleon formicarius*** Linnaeus, 1767	GR	ABW9499	1	–	–	*Euroleon nostras*	13.51
**Osmylidae**	***Osmylus fulvicephalus*** (Scopoli, 1763)	DE	AAU3322	4	0	0	*Micromus variegatus*	17.01
**Raphidiidae**	***Dichrostigma flavipes*** (Stein, 1863)	DE	ACF8053	1	–	–	*Phaeostigma notata*	12.56
	***Phaeostigma notata*** (Fabricius, 1781)	DE	ACF9144	2	0.15	0.15	*Dichrostigma flavipes*	12.56
	***Raphidia ophiopsis*** Linnaeus, 1758	DE	ACF9223	1	–	–	*Phaeostigma notata*	16.66
	***Subilla confisis*** (Stephens, 1836)	DE	ACF7187	1	–	–	*Raphidia ophiopsis*	16.94
	***Xanthostigma xanthostigma*** (Schummel, 1832)	DE	ACJ9850	1	–	–	*Phaeostigma notata*	16.44
**Sialidae**	***Sialis fuliginosa*** (F. Picet, 1836)	AT	ACF6254	1	N/A	N/A	*Sialis lutaria*	10.09
	***Sialis lutaria*** (Linnaeus, 1758)	DE	AAU3181	4	0.08	0.15	*Sialis fuliginosa*	10.09
	***Sialis nigripes*** Pictet, 1865	DE	AAV6800	2	0.46	0.46	*Sialis lutaria*	12.43
**Sisyridae**	***Sisyra nigra*** (Retzius, 1783)	DEDE	AAU3596ACE8429	21	1.71	2.65	*Sisyra terminalis*	13.63
	***Sisyra terminalis*** Curtis, 1854	DE	AAU3101	4	0.18	0.3	*Sisyra nigra*	13.63

### Laboratory procedures

A single leg was removed from each specimen and sent in 96 well plates to the Canadian Center for DNA Barcoding (CCDB) for standardized, high-throughput DNA extraction, PCR amplification and bidirectional Sanger sequencing (www.dnabarcoding.ca/pa/ge/research/protocols). The amplified target region has a length of 658 bp, starting from the 5′end of the mitochondrial *cytochrome oxidase c* (COI) gene, which includes the 648 bp barcode region [13].

### Data analysis

Sequence divergences (mean and maximum intraspecific variation and minimum genetic distance to the nearest-neighbour species) were calculated using the “*Barcode Gap Analysis*” tool on BOLD, employing the Kimura-2-Parameter distance metric and MUSCLE for sequence alignment. We only included sequences with a length of more than 500 bp in the analyses. The “*BIN Discordance*” analysis on BOLD was used to reveal species clusters which shared a BIN, and those which were assigned to two or more BINs. The Barcode Index Number (BIN) is assigned by BOLD and it represents a globally unique identifier for specimens with closely similar COI barcode sequences [14]. In most cases, members of a BIN belong to a single species recognized by traditional taxonomy [10].

## Results and Discussion

**Figure 2:**
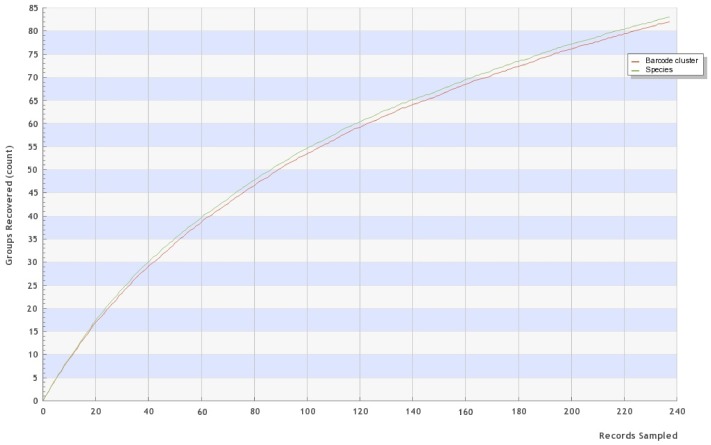
Accumulation curve for the 83 species and 82 BINs with DNA barcodes from Bavarian species. Accumulation curve (from BOLD database; randomized; 100 iterations) for the 237 barcoded individuals (>500 bp).

The successfully sequenced specimens were assigned to 83 species by morphological taxonomy while the barcode data assigned them to 82 BINs (see accumulation curve, **Fig. 2**). The 83% success rate in DNA barcode recovery was high, considering that 60% of the specimens were dry and three quarters of the other specimens had been stored, suboptimally, in 80% EtOH. Only 10% of the specimens were optimally conserved in 95% EtOH.

1. 66 of the 83 species (80%) recognized by traditional taxonomy were represented by a single BIN cluster. Each of these clusters was clearly separated from all neighboring species, meaning that the species in question can be identified unambiguously by DNA barcoding.
2. Eleven species (13%), including 4 pairs and one triplet, shared a BIN. Three of these species show constant (though small) genetic divergences from other species, meaning that they can be identified. The other eight species (four pairs) cannot be discriminated using the barcode fragment. Additional marker genes or morphological and ecological re-analysis might resolve these taxa or they may be synonyms.
3. Five species (6%) were assigned to more than one BIN. Four of these cases were assigned to two BINs, while members of the final species were placed into three BINs. Because all of these cases of additional BINs are unique, i.e. none shares its sequence with any other neuropterid species; all species in this category can be unambiguously identified. However, specimens of these species assigned to different BINs should be carefully checked for differences in morphological characters and for divergence in other marker genes to establish if they are cases of cryptic or sibling species.

### Raphidioptera (Inocellidae, Raphidiidae)

We obtained barcode sequences for 5 of the 8 species of Raphidiidae and one sequence for the only species of Inocellidae known from Bavaria [5]. All species of Inocellidae and Raphidiidae cluster together in a single clade, but the six species show a high mean interspecific divergence of 15.26%.

### Megaloptera (Sialidae)

We obtained barcodes for three of the four species of Sialidae [6]. These species were clearly separated and the mean intraspecific divergence in *S. lutaria* (Linnaeus, 1758) was low (0.08%).

### Coniopterygidae

We analyzed 14 of the 16 species of Coniopterygidae reported from Bavaria [15]. High intraspecific variation (>2%) was found in three species, cases that likely represent instances of overlooked species. Specimens of *Coniopteryx pygmaea* Enderlein, 1906 (n = 9) were assigned to three BINs (BOLD:AAU4143, BOLD:AAV8087 and BOLD:ACJ9303) with minimum pairwise distances ranging from 13.66–14.83%. Two barcode clusters were detected for both *Conwentzia psociformis* (Curtis, 1834) (n = 4) (BOLD:ACF6246, BOLD:ACJ9308) with a minimum distance of 13.28% and *Semidalis aleyrodiformis* (Stephens, 1836) (n = 7) (BOLD:AAU2412, BOLD:AAU2413) with a minimum distances of 3.77%. Two species of *Coniopteryx* (*C*. *esbenpeterseni* Tjeder, 1930, *C. lentiae* Aspöck & Aspöck, 1964) were assigned to the same BIN (BOLD:AAU4414), but possessed a pairwise distance of 1.16%, allowing their identification.

### Chrysopidae

COI barcode sequences were obtained for 25 of the 28 chrysopid species reported from Bavaria [7]. No cases of high intraspecific divergence were detected using barcode gap analysis. Three species (*C. lucasina* Lacroix, 1912, *C. pallida* Henry et al., 2002, *C. carnea* Stephens, 1836) in the *Chrysoperla carnea* group were assigned to the same BIN (BOLD:AAB0373). The first two species have very similar barcode sequences with only 0.27% of mean distance between them. *C. carnea* showed a minimum distance of 0.76% from the other two species, allowing its diagnosis. The taxonomy of the *C. carnea* group has recently been reviewed by Henry et al. [16]. Although more than 20 species can be clearly identified by their duetting behavior, separation based on 4630 bp combined mitochondrial DNA sequences from ND2, COI, COII and ND5 was not possible [17]. Thus, speciation seems to be driven by strong premating isolation within this group [17].

### Hemerobiidae

We obtained barcode sequences from 27 of the 36 hemerobiid species reported from Bavaria [7]. Barcode gap analysis on BOLD revealed three pairs of sister species which were assigned to the same BIN: *Hemerobius handschini* Tjeder, 1957 and *H. nitidulus* Fabricius, 1777 (BOLD:ABU9615), *Wesmaelius concinnus* (Stephens, 1836) and *W. quadrifasciatus* (Reuter, 1894) (BOLD:ABU9030) and *Hemerobius atrifrons McLachlan, 1868* and *H. contumax* Tjeder, 1932 (BOLD:ACF6575). *H. handschini* and *H. nitidulus* may be diagnosed by barcodes, but this needs confirmation as the present analysis revealed just a single diagnostic difference (0.15% divergence). These species, together with *H. schedlii* are closely related and their validity has been questioned by Aspöck et al. [12] considering the high variability of morphological characters. No interspecific divergence was detected for the other two species pairs *W. concinnus/W. quadrifasciatus and H. atrifrons/H. contumax*. The members of these two species pairs generally show morphological differences [12], but Monserrat [18] noted problems in the discrimination of *H.atrifrons* and *H. contumax*. Additional genetic data should be obtained to validate the status of these taxa.

COI data suggest that within the genus *Sympherobius* Banks, 1904 the subgenera *Sympherobius* s.str Banks, 1904 (*S. pygmaeus* (Rambur, 1842), *S. elegans* (Stephens, 1836)) and *Niremberge* Navas 1909 (*S. fuscescens* (Wallengren, 1863), *S. pellucidus (*Walker, 1853), and *S. klapaleki Zeleny, 1963*) are not closely related to each other (cf. Neighbor Joining Tree, **Fig. S2**, but this needs to be corroborated with additional marker genes. *S. pygmaeus* (n = 3) splits into two BINs (BOLD:ACF9381, BOLD:ACG0292) with a minimum distances of 3.12%, suggesting that it may be a sibling species pair.

### Sisyridae, Osmylidae, Myrmeleontidae and Ascalaphidae

We obtained COI sequences for 2 of 3 species of Sisyridae from Bavaria [7]. DNA barcode analysis revealed that *Sisyra nigra* (Retzius, 1783) (n = 3) includes two BINs (BOLD:AAU3596, BOLD:ACE8429) with a minimum distances of 2.65%. For the remaining families of Neuropterida, the analysis did not recover any case of barcode sharing or potential cryptic diversity. We obtained barcode sequences for the only species of Osmylidae species, for all four species of Myrmelontidae and for one of the two species of Ascalaphidae.

## Concluding Remarks

Except for a few cases of BIN sharing and other cases of deep divergence that may reflect cryptic diversity, the present COI barcode data allow unambiguous identification of 75/83 (90%) of the species of Bavarian Neuropterida species which were examined. Furthermore, one or more of the four species pairs that could not be separated may represent cases of unrecognized synonymy. Interestingly, the interspecific distances (to the nearest neighbours) within the Chrysopidae were considerably lower (5–10%) than those in the Coniopterygidae, Hemerobiidae, and Myrmeleontidae (10–20%).

The success rate (83%) in obtaining DNA barcodes was high, especially considering the fact that most specimens were stored under suboptimal conditions. Thus dried specimens of Neuropterida are a suitable source for DNA barcoding as is also the case for Lepidoptera [9], [10], [13], [19], while other groups, such as Coleoptera, are more problematic [20].

## Supporting Information

Figure S1 **Taxon ID Tree (established in BOLD) – BIN clusters appear in different colours.**
(PDF)

Figure S2 **Neighbor joining tree of the genus** ***Sympherobius*** **(established in BOLD) – BIN clusters appear in different colours.**
(PDF)

Appendix S1 **List of all specimens used in this study, including BOLD process IDs, BOLD sample IDs and Genbank accession numbers.**
(DOCX)
