# Supplementary figures and images for: Barcoding Fauna Bavarica: 78% of the Neuropterida Fauna Barcoded!
Source: PLoS One. 2014 Oct 6;9(10):e109719. doi: 10.1371/journal.pone.0109719 (PMC4186837; doi:10.1371/journal.pone.0109719)

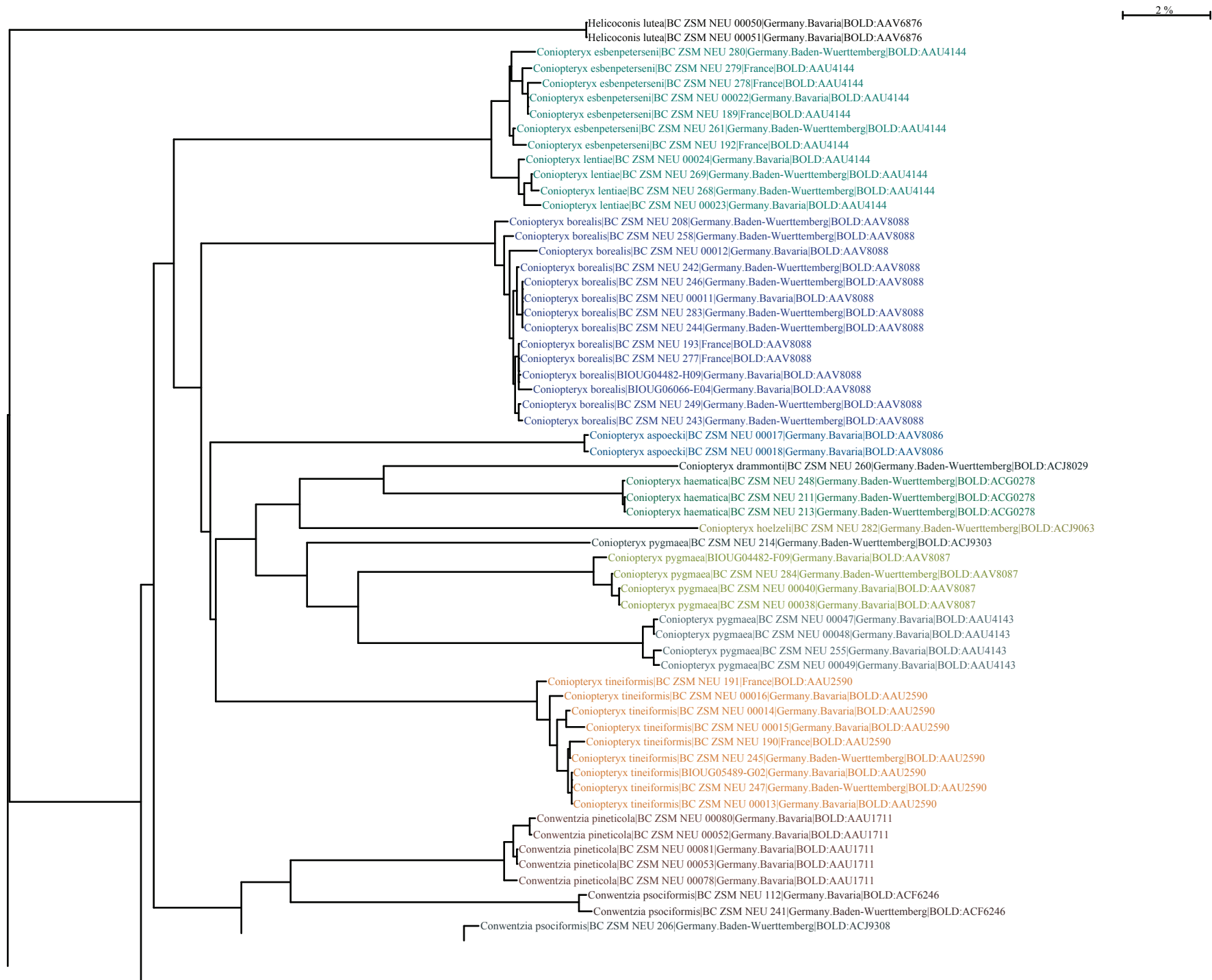

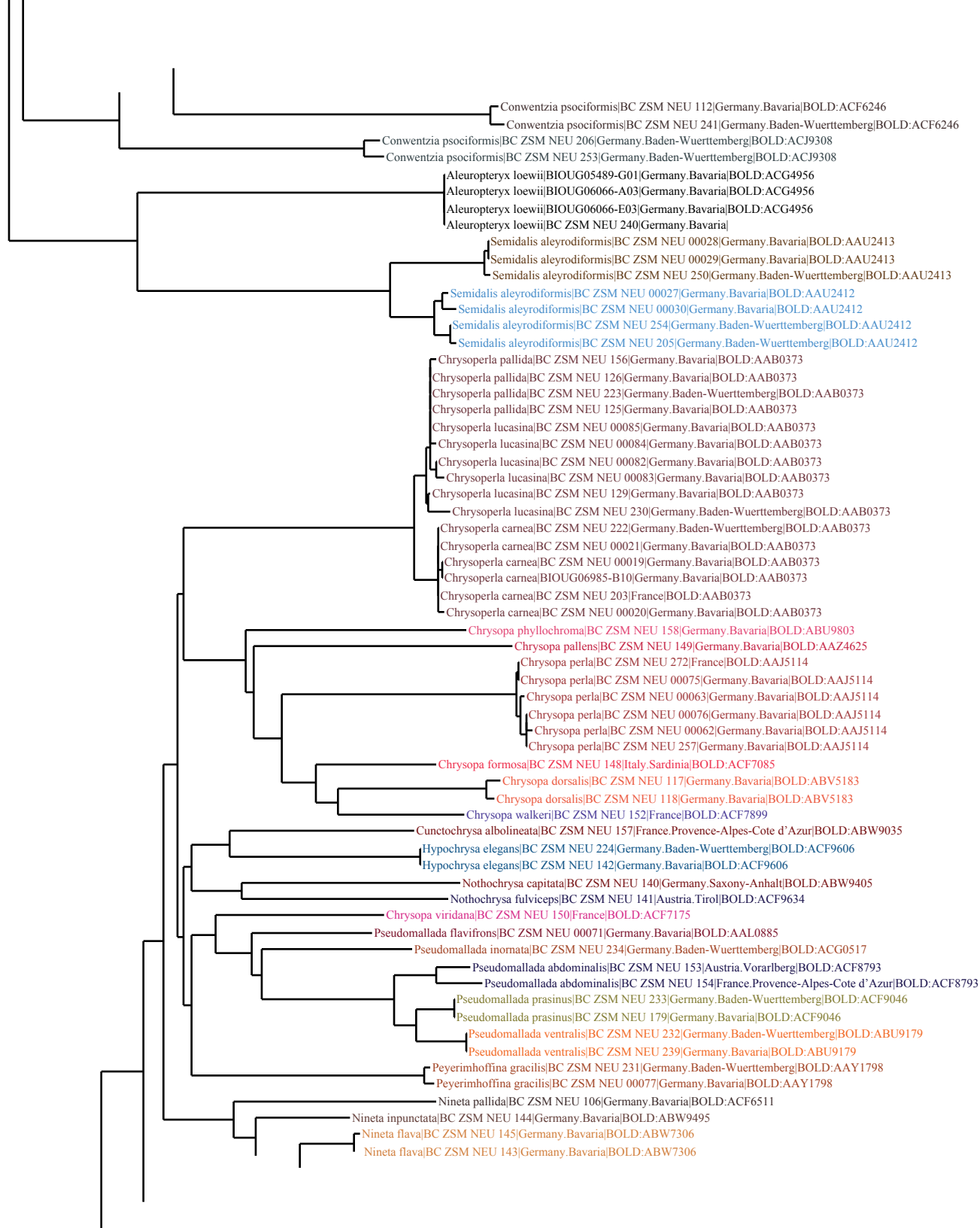

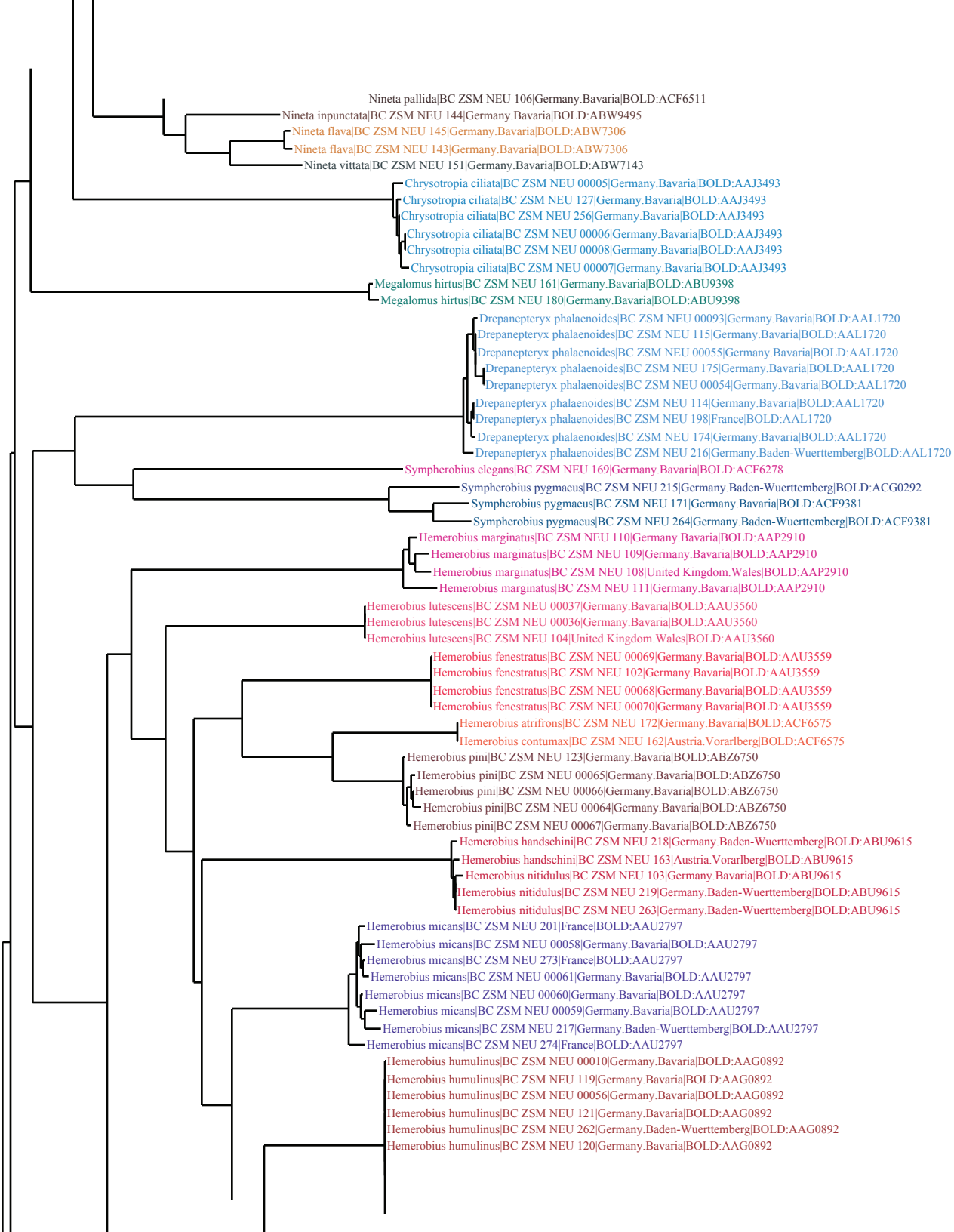

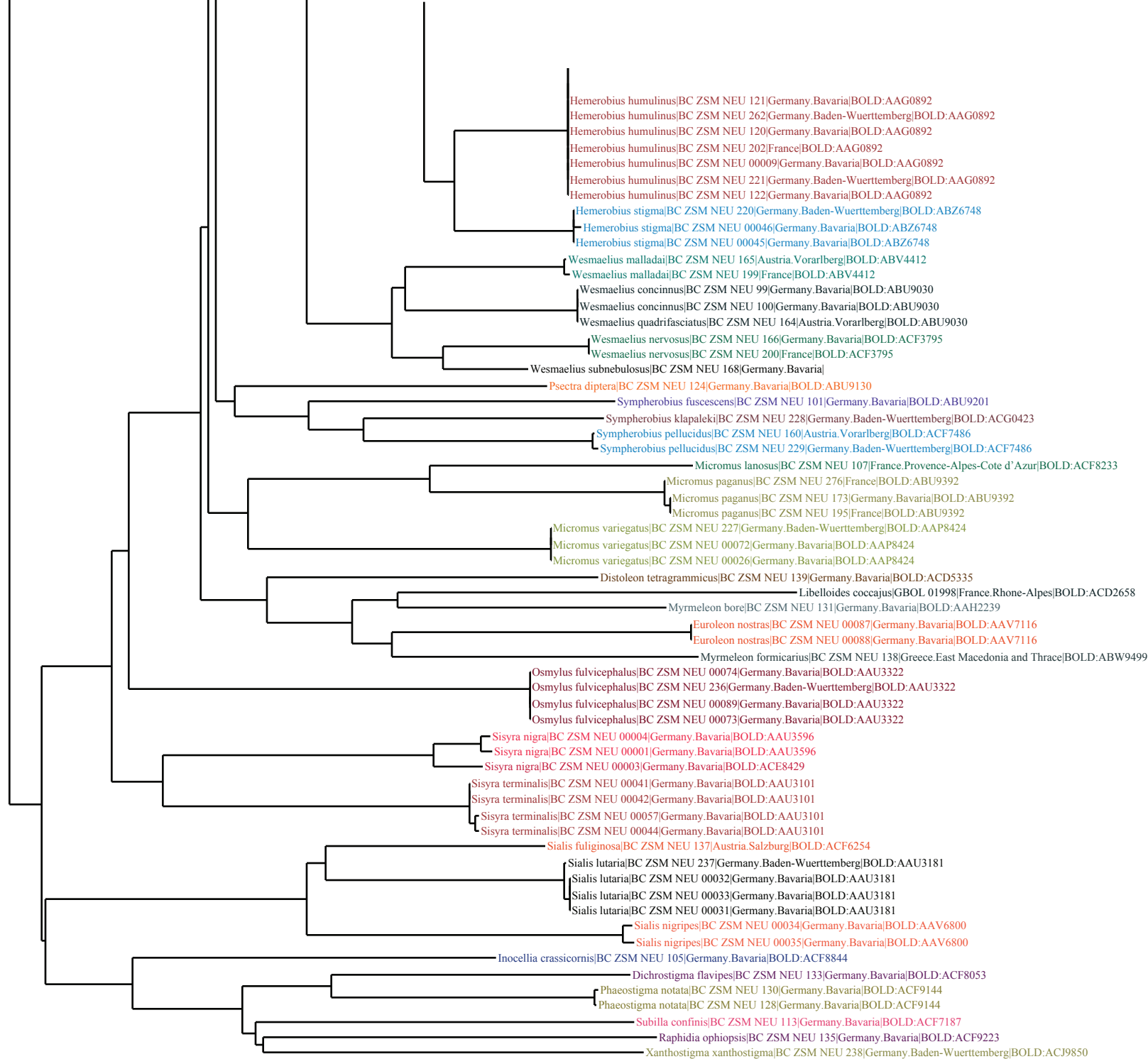

Supplement: Figure S1 — Taxon ID Tree (established in BOLD) – BIN clusters appear in different colours. (PDF) [file pone.0109719.s001.pdf]
