# Supplementary material for: Barcoding Fauna Bavarica: 78% of the Neuropterida Fauna Barcoded!
Source: PLoS One. 2014 Oct 6;9(10):e109719. doi: 10.1371/journal.pone.0109719 (PMC4186837; doi:10.1371/journal.pone.0109719)
Supplement: Figure S2 — Neighbor joining tree of the genus Sympherobius (established in BOLD) – BIN clusters appear in different colours. (PDF) [file pone.0109719.s002.pdf]

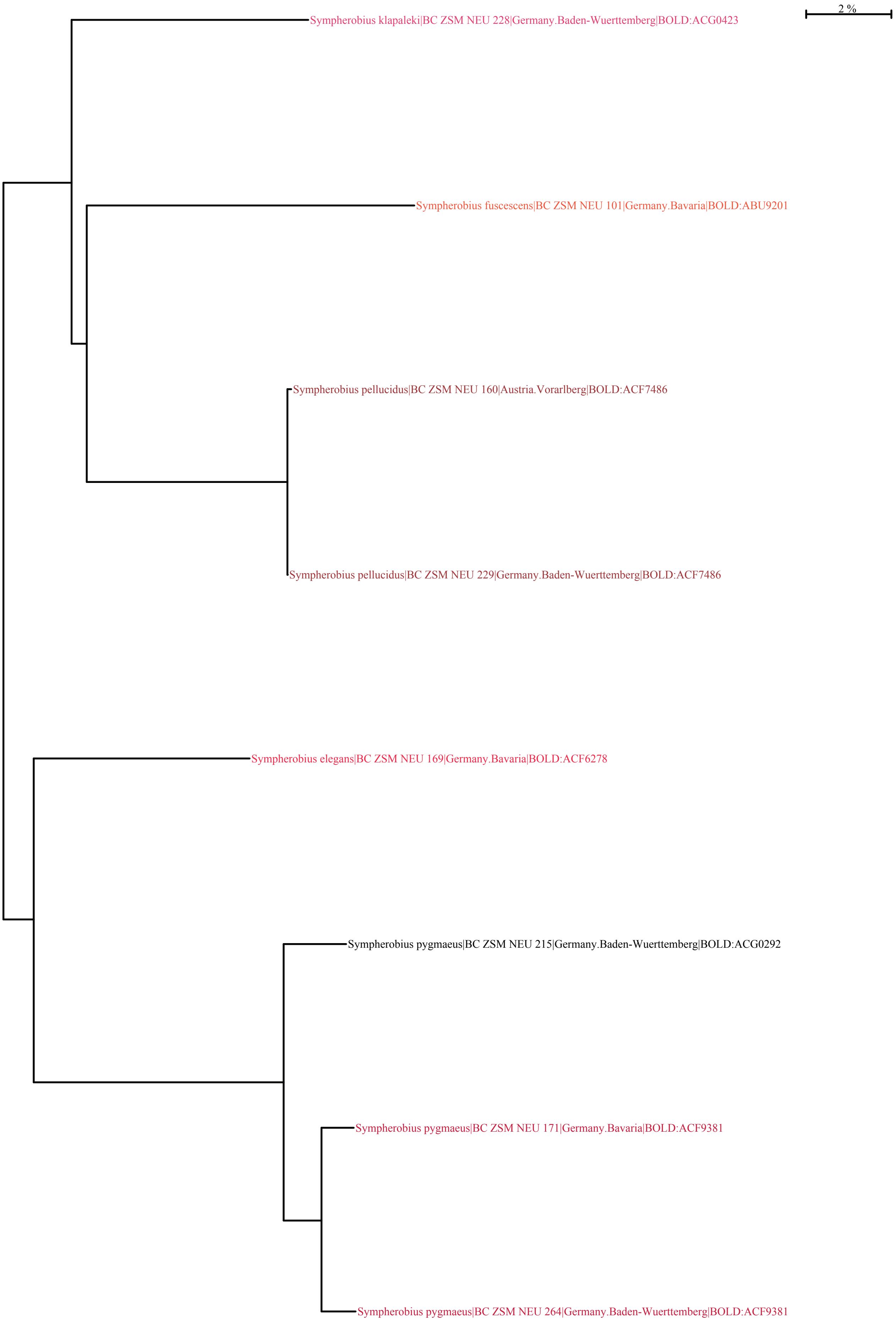
2 %
Sympherobius klapaleki|BC ZSM NEU 228|Germany.Baden-Wuerttemberg|BOLD:ACG0423
Sympherobius fuscescens|BC ZSM NEU 101|Germany.Bavaria|BOLD:ABU9201
Sympherobius pellucidus|BC ZSM NEU 160|Austria.Vorarlberg|BOLD:ACF7486
Sympherobius pellucidus|BC ZSM NEU 229|Germany.Baden-Wuerttemberg|BOLD:ACF7486
Sympherobius elegans|BC ZSM NEU 169|Germany.Bavaria|BOLD:ACF6278
Sympherobius pygmaeus|BC ZSM NEU 215|Germany.Baden-Wuerttemberg|BOLD:ACG0292
Sympherobius pygmaeus|BC ZSM NEU 171|Germany.Bavaria|BOLD:ACF9381
Sympherobius pygmaeus|BC ZSM NEU 264|Germany.Baden-Wuerttemberg|BOLD:ACF9381
