# Supplementary material for: Barcoding Fauna Bavarica: 78% of the Neuropterida Fauna Barcoded!
Source: PLoS One. 2014 Oct 6;9(10):e109719. doi: 10.1371/journal.pone.0109719 (PMC4186837; doi:10.1371/journal.pone.0109719)
Supplement: Appendix S1 — List of all specimens used in this study, including BOLD process IDs, BOLD sample IDs and Genbank accession numbers. (DOCX) [file pone.0109719.s003.docx]

| **Identification** | **Process ID** | **Sample ID** | **COI-5P Accession** |
| --- | --- | --- | --- |
| Aleuropteryx loewii | GMGRE2894-13 | BIOUG06066-E03 | KJ592464 |
| Aleuropteryx loewii | FBNE098-13 | BC ZSM NEU 98 |  |
| Aleuropteryx loewii | GMGRD1567-13 | BIOUG05489-G01 | KJ592513 |
| Aleuropteryx loewii | GMGRD3681-13 | BIOUG06066-A03 | KJ592488 |
| Aleuropteryx loewii | FBNE240-13 | BC ZSM NEU 240 | KJ592463 |
| Chrysopa abbreviata | FBNE146-13 | BC ZSM NEU 146 |  |
| Chrysopa commata | FBNE147-13 | BC ZSM NEU 147 |  |
| Chrysopa dorsalis | FBNE117-13 | BC ZSM NEU 117 | KJ592482 |
| Chrysopa dorsalis | FBNE118-13 | BC ZSM NEU 118 | KJ592514 |
| Chrysopa formosa | FBNE148-13 | BC ZSM NEU 148 | KJ592501 |
| Chrysopa pallens | FBNE149-13 | BC ZSM NEU 149 | KJ592516 |
| Chrysopa perla | FBNE092-11 | BC ZSM NEU 00092 |  |
| Chrysopa perla | FBNE075-11 | BC ZSM NEU 00075 | JN299370 |
| Chrysopa perla | FBNE076-11 | BC ZSM NEU 00076 | JN299371 |
| Chrysopa perla | FBNE063-11 | BC ZSM NEU 00063 | JN299358 |
| Chrysopa perla | FBNE272-13 | BC ZSM NEU 272 | KJ592478 |
| Chrysopa perla | FBNE204-13 | BC ZSM NEU 204 |  |
| Chrysopa perla | FBNE062-11 | BC ZSM NEU 00062 | JN299357 |
| Chrysopa perla | FBNE257-13 | BC ZSM NEU 257 | KJ592454 |
| Chrysopa phyllochroma | FBNE158-13 | BC ZSM NEU 158 | KJ592436 |
| Chrysopa viridana | FBNE150-13 | BC ZSM NEU 150 | KJ592565 |
| Chrysopa walkeri | FBNE152-13 | BC ZSM NEU 152 | KJ592546 |
| Chrysoperla carnea | FBNE021-11 | BC ZSM NEU 00021 | JN299320 |
| Chrysoperla carnea | FBNE222-13 | BC ZSM NEU 222 | KJ592518 |
| Chrysoperla carnea | FBNE019-11 | BC ZSM NEU 00019 | JN299318 |
| Chrysoperla carnea | GMGRF5357-13 | BIOUG06985-B10 | KJ592434 |
| Chrysoperla carnea | FBNE203-13 | BC ZSM NEU 203 | KJ592484 |
| Chrysoperla carnea | FBNE020-11 | BC ZSM NEU 00020 | JN299319 |
| Chrysoperla lucasina | FBNE083-11 | BC ZSM NEU 00083 | JN299375 |
| Chrysoperla lucasina | FBNE085-11 | BC ZSM NEU 00085 | JN299377 |
| Chrysoperla lucasina | FBNE129-13 | BC ZSM NEU 129 | KJ592560 |
| Chrysoperla lucasina | FBNE230-13 | BC ZSM NEU 230 | KJ592575 |
| Chrysoperla lucasina | FBNE084-11 | BC ZSM NEU 00084 | JN299376 |
| Chrysoperla lucasina | FBNE082-11 | BC ZSM NEU 00082 | KJ592506 |
| Chrysoperla pallida | FBNE156-13 | BC ZSM NEU 156 | KJ592477 |
| Chrysoperla pallida | FBNE223-13 | BC ZSM NEU 223 | KJ592534 |
| Chrysoperla pallida | FBNE126-13 | BC ZSM NEU 126 | KJ592554 |
| Chrysoperla pallida | FBNE125-13 | BC ZSM NEU 125 | KJ592562 |
| Chrysotropia ciliata | FBNE007-11 | BC ZSM NEU 00007 | JN299306 |
| Chrysotropia ciliata | FBNE005-11 | BC ZSM NEU 00005 | JN299304 |
| Chrysotropia ciliata | FBNE008-11 | BC ZSM NEU 00008 | JN299307 |
| Chrysotropia ciliata | FBNE235-13 | BC ZSM NEU 235 | KJ592443 |
| Chrysotropia ciliata | FBNE006-11 | BC ZSM NEU 00006 | JN299305 |
| Chrysotropia ciliata | FBNE127-13 | BC ZSM NEU 127 | KJ592511 |
| Chrysotropia ciliata | FBNE256-13 | BC ZSM NEU 256 | KJ592583 |
| Coniopteryx | FBNE207-13 | BC ZSM NEU 207 |  |
| Coniopteryx | FBNE281-13 | BC ZSM NEU 281 |  |
| Coniopteryx aspoecki | FBNE017-11 | BC ZSM NEU 00017 | JN299316 |
| Coniopteryx aspoecki | FBNE018-11 | BC ZSM NEU 00018 | JN299317 |
| Coniopteryx borealis | FBNE249-13 | BC ZSM NEU 249 | KJ592430 |
| Coniopteryx borealis | GMGRC2195-13 | BIOUG04482-H09 | KJ592508 |
| Coniopteryx borealis | FBNE246-13 | BC ZSM NEU 246 | KJ592538 |
| Coniopteryx borealis | FBNE258-13 | BC ZSM NEU 258 | KJ592539 |
| Coniopteryx borealis | FBNE242-13 | BC ZSM NEU 242 | KJ592517 |
| Coniopteryx borealis | FBNE193-13 | BC ZSM NEU 193 | KJ592480 |
| Coniopteryx borealis | FBNE208-13 | BC ZSM NEU 208 | KJ592515 |
| Coniopteryx borealis | FBNE012-11 | BC ZSM NEU 00012 | JN299311 |
| Coniopteryx borealis | GMGRE2895-13 | BIOUG06066-E04 | KJ592502 |
| Coniopteryx borealis | FBNE011-11 | BC ZSM NEU 00011 | JN299310 |
| Coniopteryx borealis | FBNE277-13 | BC ZSM NEU 277 | KJ592469 |
| Coniopteryx borealis | FBNE243-13 | BC ZSM NEU 243 | KJ592547 |
| Coniopteryx borealis | FBNE283-13 | BC ZSM NEU 283 | KJ592528 |
| Coniopteryx borealis | FBNE244-13 | BC ZSM NEU 244 | KJ592529 |
| Coniopteryx drammonti | FBNE210-13 | BC ZSM NEU 210 |  |
| Coniopteryx drammonti | FBNE260-13 | BC ZSM NEU 260 | KJ592587 |
| Coniopteryx drammonti | FBNE267-13 | BC ZSM NEU 267 |  |
| Coniopteryx esbenpeterseni | FBNE189-13 | BC ZSM NEU 189 | KJ592494 |
| Coniopteryx esbenpeterseni | FBNE279-13 | BC ZSM NEU 279 | KJ592556 |
| Coniopteryx esbenpeterseni | FBNE280-13 | BC ZSM NEU 280 | KJ592431 |
| Coniopteryx esbenpeterseni | FBNE278-13 | BC ZSM NEU 278 | KJ592588 |
| Coniopteryx esbenpeterseni | FBNE192-13 | BC ZSM NEU 192 | KJ592509 |
| Coniopteryx esbenpeterseni | FBNE261-13 | BC ZSM NEU 261 | KJ592577 |
| Coniopteryx esbenpeterseni | FBNE022-11 | BC ZSM NEU 00022 | JN299321 |
| Coniopteryx haematica | FBNE211-13 | BC ZSM NEU 211 | KJ592440 |
| Coniopteryx haematica | FBNE259-13 | BC ZSM NEU 259 |  |
| Coniopteryx haematica | FBNE097-13 | BC ZSM NEU 97 |  |
| Coniopteryx haematica | FBNE248-13 | BC ZSM NEU 248 | KJ592452 |
| Coniopteryx haematica | FBNE213-13 | BC ZSM NEU 213 | KJ592530 |
| Coniopteryx hoelzeli | FBNE282-13 | BC ZSM NEU 282 | KJ592453 |
| Coniopteryx lentiae | FBNE270-13 | BC ZSM NEU 270 |  |
| Coniopteryx lentiae | FBNE023-11 | BC ZSM NEU 00023 | JN299322 |
| Coniopteryx lentiae | FBNE269-13 | BC ZSM NEU 269 | KJ592586 |
| Coniopteryx lentiae | FBNE268-13 | BC ZSM NEU 268 | KJ592461 |
| Coniopteryx lentiae | FBNE024-11 | BC ZSM NEU 00024 | JN299323 |
| Coniopteryx lentiae | FBNE209-13 | BC ZSM NEU 209 |  |
| Coniopteryx pygmaea | FBNE048-11 | BC ZSM NEU 00048 | JN299343 |
| Coniopteryx pygmaea | FBNE255-13 | BC ZSM NEU 255 | KJ592568 |
| Coniopteryx pygmaea | FBNE047-11 | BC ZSM NEU 00047 | JN299342 |
| Coniopteryx pygmaea | FBNE038-11 | BC ZSM NEU 00038 | JN299336 |
| Coniopteryx pygmaea | FBNE265-13 | BC ZSM NEU 265 |  |
| Coniopteryx pygmaea | FBNE039-11 | BC ZSM NEU 00039 |  |
| Coniopteryx pygmaea | FBNE214-13 | BC ZSM NEU 214 | KJ592561 |
| Coniopteryx pygmaea | GMGRC2171-13 | BIOUG04482-F09 | KJ592475 |
| Coniopteryx pygmaea | FBNE284-13 | BC ZSM NEU 284 | KJ592555 |
| Coniopteryx pygmaea | FBNE040-11 | BC ZSM NEU 00040 | JN299337 |
| Coniopteryx pygmaea | FBNE049-11 | BC ZSM NEU 00049 | JN299344 |
| Coniopteryx tineiformis | FBNE013-11 | BC ZSM NEU 00013 | JN299312 |
| Coniopteryx tineiformis | FBNE245-13 | BC ZSM NEU 245 | KJ592451 |
| Coniopteryx tineiformis | FBNE212-13 | BC ZSM NEU 212 |  |
| Coniopteryx tineiformis | FBNE015-11 | BC ZSM NEU 00015 | JN299314 |
| Coniopteryx tineiformis | FBNE014-11 | BC ZSM NEU 00014 | JN299313 |
| Coniopteryx tineiformis | FBNE016-11 | BC ZSM NEU 00016 | JN299315 |
| Coniopteryx tineiformis | FBNE266-13 | BC ZSM NEU 266 |  |
| Coniopteryx tineiformis | FBNE247-13 | BC ZSM NEU 247 | KJ592441 |
| Coniopteryx tineiformis | FBNE190-13 | BC ZSM NEU 190 | KJ592553 |
| Coniopteryx tineiformis | FBNE191-13 | BC ZSM NEU 191 | KJ592541 |
| Coniopteryx tineiformis | GMGRD1568-13 | BIOUG05489-G02 | KJ592537 |
| Conwentzia pineticola | FBNE081-11 | BC ZSM NEU 00081 | JN299374 |
| Conwentzia pineticola | FBNE080-11 | BC ZSM NEU 00080 | JN299373 |
| Conwentzia pineticola | FBNE078-11 | BC ZSM NEU 00078 | JN299372 |
| Conwentzia pineticola | FBNE052-11 | BC ZSM NEU 00052 | JN299347 |
| Conwentzia pineticola | FBNE053-11 | BC ZSM NEU 00053 | JN299348 |
| Conwentzia pineticola | FBNE079-11 | BC ZSM NEU 00079 |  |
| Conwentzia psociformis | FBNE206-13 | BC ZSM NEU 206 | KJ592525 |
| Conwentzia psociformis | FBNE253-13 | BC ZSM NEU 253 | KJ592573 |
| Conwentzia psociformis | FBNE112-13 | BC ZSM NEU 112 | KJ592503 |
| Conwentzia psociformis | FBNE251-13 | BC ZSM NEU 251 |  |
| Conwentzia psociformis | FBNE241-13 | BC ZSM NEU 241 | KJ592483 |
| Conwentzia psociformis | FBNE271-13 | BC ZSM NEU 271 |  |
| Cunctochrysa albolineata | FBNE157-13 | BC ZSM NEU 157 | KJ592467 |
| Dichrostigma flavipes | FBNE133-13 | BC ZSM NEU 133 | KJ592551 |
| Distoleon tetragrammicus | FBNE139-13 | BC ZSM NEU 139 | KJ592486 |
| Drepanepteryx phalaenoides | FBNE174-13 | BC ZSM NEU 174 | KJ592535 |
| Drepanepteryx phalaenoides | FBNE054-11 | BC ZSM NEU 00054 | JN299349 |
| Drepanepteryx phalaenoides | FBNE216-13 | BC ZSM NEU 216 | KJ592432 |
| Drepanepteryx phalaenoides | FBNE198-13 | BC ZSM NEU 198 | KJ592471 |
| Drepanepteryx phalaenoides | FBNE055-11 | BC ZSM NEU 00055 | JN299350 |
| Drepanepteryx phalaenoides | FBNE114-13 | BC ZSM NEU 114 | KJ592504 |
| Drepanepteryx phalaenoides | FBNE175-13 | BC ZSM NEU 175 | KJ592526 |
| Drepanepteryx phalaenoides | FBNE115-13 | BC ZSM NEU 115 | KJ592569 |
| Drepanepteryx phalaenoides | FBNE093-11 | BC ZSM NEU 00093 | JN299381 |
| Euroleon nostras | FBNE088-11 | BC ZSM NEU 00088 | JN299379 |
| Euroleon nostras | FBNE087-11 | BC ZSM NEU 00087 | JN299378 |
| Helicoconis lutea | FBNE050-11 | BC ZSM NEU 00050 | JN299345 |
| Helicoconis lutea | FBNE051-11 | BC ZSM NEU 00051 | JN299346 |
| Hemerobius atrifrons | FBNE172-13 | BC ZSM NEU 172 | KJ592499 |
| Hemerobius contumax | FBNE162-13 | BC ZSM NEU 162 | KJ592437 |
| Hemerobius fenestratus | FBNE102-13 | BC ZSM NEU 102 | KJ592521 |
| Hemerobius fenestratus | FBNE069-11 | BC ZSM NEU 00069 | JN299364 |
| Hemerobius fenestratus | FBNE070-11 | BC ZSM NEU 00070 | JN299365 |
| Hemerobius fenestratus | FBNE068-11 | BC ZSM NEU 00068 | JN299363 |
| Hemerobius handschini | FBNE218-13 | BC ZSM NEU 218 | KJ592566 |
| Hemerobius handschini | FBNE163-13 | BC ZSM NEU 163 | KJ592458 |
| Hemerobius humulinus | FBNE122-13 | BC ZSM NEU 122 | KJ592446 |
| Hemerobius humulinus | FBNE202-13 | BC ZSM NEU 202 | KJ592457 |
| Hemerobius humulinus | FBNE121-13 | BC ZSM NEU 121 | KJ592505 |
| Hemerobius humulinus | FBNE119-13 | BC ZSM NEU 119 | KJ592456 |
| Hemerobius humulinus | FBNE010-11 | BC ZSM NEU 00010 | JN299309 |
| Hemerobius humulinus | FBNE009-11 | BC ZSM NEU 00009 | JN299308 |
| Hemerobius humulinus | FBNE056-11 | BC ZSM NEU 00056 | JN299351 |
| Hemerobius humulinus | FBNE120-13 | BC ZSM NEU 120 | KJ592460 |
| Hemerobius humulinus | FBNE221-13 | BC ZSM NEU 221 | KJ592500 |
| Hemerobius humulinus | FBNE262-13 | BC ZSM NEU 262 | KJ592473 |
| Hemerobius lutescens | FBNE104-13 | BC ZSM NEU 104 | KJ592490 |
| Hemerobius lutescens | FBNE036-11 | BC ZSM NEU 00036 | JN299334 |
| Hemerobius lutescens | FBNE037-11 | BC ZSM NEU 00037 | JN299335 |
| Hemerobius marginatus | FBNE111-13 | BC ZSM NEU 111 | KJ592540 |
| Hemerobius marginatus | FBNE110-13 | BC ZSM NEU 110 | KJ592447 |
| Hemerobius marginatus | FBNE108-13 | BC ZSM NEU 108 | KJ592574 |
| Hemerobius marginatus | FBNE109-13 | BC ZSM NEU 109 | KJ592489 |
| Hemerobius micans | FBNE060-11 | BC ZSM NEU 00060 | JN299355 |
| Hemerobius micans | FBNE217-13 | BC ZSM NEU 217 | KJ592545 |
| Hemerobius micans | FBNE061-11 | BC ZSM NEU 00061 | JN299356 |
| Hemerobius micans | FBNE059-11 | BC ZSM NEU 00059 | JN299354 |
| Hemerobius micans | FBNE201-13 | BC ZSM NEU 201 | KJ592558 |
| Hemerobius micans | FBNE058-11 | BC ZSM NEU 00058 | JN299353 |
| Hemerobius micans | FBNE274-13 | BC ZSM NEU 274 | KJ592442 |
| Hemerobius micans | FBNE273-13 | BC ZSM NEU 273 | KJ592544 |
| Hemerobius nitidulus | FBNE103-13 | BC ZSM NEU 103 | KJ592468 |
| Hemerobius nitidulus | FBNE219-13 | BC ZSM NEU 219 | KJ592435 |
| Hemerobius nitidulus | FBNE263-13 | BC ZSM NEU 263 | KJ592564 |
| Hemerobius pini | FBNE064-11 | BC ZSM NEU 00064 | JN299359 |
| Hemerobius pini | FBNE066-11 | BC ZSM NEU 00066 | JN299361 |
| Hemerobius pini | FBNE067-11 | BC ZSM NEU 00067 | JN299362 |
| Hemerobius pini | FBNE123-13 | BC ZSM NEU 123 | KJ592459 |
| Hemerobius pini | FBNE065-11 | BC ZSM NEU 00065 | JN299360 |
| Hemerobius stigma | FBNE045-11 | BC ZSM NEU 00045 | JN299341 |
| Hemerobius stigma | FBNE046-11 | BC ZSM NEU 00046 | KJ592496 |
| Hemerobius stigma | FBNE220-13 | BC ZSM NEU 220 | KJ592498 |
| Hypochrysa elegans | FBNE224-13 | BC ZSM NEU 224 | KJ592497 |
| Hypochrysa elegans | FBNE142-13 | BC ZSM NEU 142 | KJ592543 |
| Inocellia crassicornis | FBNE105-13 | BC ZSM NEU 105 | KJ592491 |
| Libelloides coccajus | FBNE178-13 | BC ZSM NEU 178 |  |
| Libelloides coccajus | FBNE177-13 | BC ZSM NEU 177 |  |
| Libelloides coccajus | GBCOU380-13 | GBOL 01998 | KJ592570 |
| Megalomus hirtus | FBNE161-13 | BC ZSM NEU 161 | KJ592550 |
| Megalomus hirtus | FBNE180-13 | BC ZSM NEU 180 | KJ592519 |
| Micromus angulatus | FBNE086-11 | BC ZSM NEU 00086 | KJ592450 |
| Micromus angulatus | FBNE285-13 | BC ZSM NEU 285 | KJ592567 |
| Micromus angulatus | FBNE196-13 | BC ZSM NEU 196 |  |
| Micromus angulatus | FBNE226-13 | BC ZSM NEU 226 |  |
| Micromus lanosus | FBNE194-13 | BC ZSM NEU 194 |  |
| Micromus lanosus | FBNE107-13 | BC ZSM NEU 107 | KJ592549 |
| Micromus paganus | FBNE276-13 | BC ZSM NEU 276 | KJ592433 |
| Micromus paganus | FBNE195-13 | BC ZSM NEU 195 | KJ592581 |
| Micromus paganus | FBNE173-13 | BC ZSM NEU 173 | KJ592495 |
| Micromus variegatus | FBNE197-13 | BC ZSM NEU 197 |  |
| Micromus variegatus | FBNE072-11 | BC ZSM NEU 00072 | JN299367 |
| Micromus variegatus | FBNE025-11 | BC ZSM NEU 00025 | KJ592523 |
| Micromus variegatus | FBNE090-11 | BC ZSM NEU 00090 |  |
| Micromus variegatus | FBNE026-11 | BC ZSM NEU 00026 | JN299324 |
| Micromus variegatus | FBNE227-13 | BC ZSM NEU 227 | KJ592559 |
| Micromus variegatus | FBNE275-13 | BC ZSM NEU 275 |  |
| Myrmeleon bore | FBNE131-13 | BC ZSM NEU 131 | KJ592439 |
| Myrmeleon formicarius | FBNE138-13 | BC ZSM NEU 138 | KJ592531 |
| Neuroptera | GMGRD3468-13 | BIOUG03760-F01 |  |
| Neuroptera | GBCOU1235-13 | GBOL01428 |  |
| Nineta flava | FBNE145-13 | BC ZSM NEU 145 | KJ592542 |
| Nineta flava | FBNE143-13 | BC ZSM NEU 143 | KJ592462 |
| Nineta inpunctata | FBNE144-13 | BC ZSM NEU 144 | KJ592571 |
| Nineta pallida | FBNE106-13 | BC ZSM NEU 106 | KJ592563 |
| Nineta vittata | FBNE091-11 | BC ZSM NEU 00091 |  |
| Nineta vittata | FBNE151-13 | BC ZSM NEU 151 | KJ592520 |
| Nothochrysa capitata | FBNE140-13 | BC ZSM NEU 140 | KJ592448 |
| Nothochrysa fulviceps | FBNE141-13 | BC ZSM NEU 141 | KJ592585 |
| Osmylus fulvicephalus | FBNE236-13 | BC ZSM NEU 236 | KJ592533 |
| Osmylus fulvicephalus | FBNE089-11 | BC ZSM NEU 00089 | JN299380 |
| Osmylus fulvicephalus | FBNE074-11 | BC ZSM NEU 00074 | JN299369 |
| Osmylus fulvicephalus | FBNE073-11 | BC ZSM NEU 00073 | JN299368 |
| Peyerimhoffina gracilis | FBNE077-11 | BC ZSM NEU 00077 | KJ592493 |
| Peyerimhoffina gracilis | FBNE231-13 | BC ZSM NEU 231 | KJ592481 |
| Phaeostigma major | FBNE132-13 | BC ZSM NEU 132 |  |
| Phaeostigma notata | FBNE130-13 | BC ZSM NEU 130 | KJ592548 |
| Phaeostigma notata | FBNE116-13 | BC ZSM NEU 116 |  |
| Phaeostigma notata | FBNE225-13 | BC ZSM NEU 225 |  |
| Phaeostigma notata | FBNE128-13 | BC ZSM NEU 128 | KJ592465 |
| Phaeostigma notata | FBNE095-11 | BC ZSM NEU 00095 |  |
| Psectra diptera | FBNE124-13 | BC ZSM NEU 124 | KJ592522 |
| Pseudomallada abdominalis | FBNE154-13 | BC ZSM NEU 154 | KJ592466 |
| Pseudomallada abdominalis | FBNE153-13 | BC ZSM NEU 153 | KJ592487 |
| Pseudomallada flavifrons | FBNE071-11 | BC ZSM NEU 00071 | JN299366 |
| Pseudomallada inornata | FBNE234-13 | BC ZSM NEU 234 | KJ592479 |
| Pseudomallada prasinus | FBNE233-13 | BC ZSM NEU 233 | KJ592536 |
| Pseudomallada prasinus | FBNE179-13 | BC ZSM NEU 179 | KJ592524 |
| Pseudomallada ventralis | FBNE239-13 | BC ZSM NEU 239 | KJ592449 |
| Pseudomallada ventralis | FBNE155-13 | BC ZSM NEU 155 |  |
| Pseudomallada ventralis | FBNE232-13 | BC ZSM NEU 232 | KJ592470 |
| Puncha ratzeburgi | FBNE136-13 | BC ZSM NEU 136 |  |
| Raphidia ophiopsis | FBNE135-13 | BC ZSM NEU 135 | KJ592579 |
| Semidalis aleyrodiformis | FBNE205-13 | BC ZSM NEU 205 | KJ592584 |
| Semidalis aleyrodiformis | FBNE028-11 | BC ZSM NEU 00028 | JN299326 |
| Semidalis aleyrodiformis | FBNE027-11 | BC ZSM NEU 00027 | JN299325 |
| Semidalis aleyrodiformis | FBNE250-13 | BC ZSM NEU 250 | KJ592510 |
| Semidalis aleyrodiformis | FBNE030-11 | BC ZSM NEU 00030 | JN299328 |
| Semidalis aleyrodiformis | FBNE029-11 | BC ZSM NEU 00029 | JN299327 |
| Semidalis aleyrodiformis | FBNE254-13 | BC ZSM NEU 254 | KJ592552 |
| Semidalis pseudouncinata | FBNE096-13 | BC ZSM NEU 96 |  |
| Sialis fuliginosa | FBNE137-13 | BC ZSM NEU 137 | KJ592429 |
| Sialis lutaria | FBNE031-11 | BC ZSM NEU 00031 | JN299329 |
| Sialis lutaria | FBNE033-11 | BC ZSM NEU 00033 | JN299331 |
| Sialis lutaria | FBNE032-11 | BC ZSM NEU 00032 | JN299330 |
| Sialis lutaria | FBNE237-13 | BC ZSM NEU 237 | KJ592532 |
| Sialis nigripes | FBNE035-11 | BC ZSM NEU 00035 | JN299333 |
| Sialis nigripes | FBNE034-11 | BC ZSM NEU 00034 | JN299332 |
| Sisyra nigra | FBNE002-11 | BC ZSM NEU 00002 |  |
| Sisyra nigra | FBNE004-11 | BC ZSM NEU 00004 | JN299303 |
| Sisyra nigra | FBNE003-11 | BC ZSM NEU 00003 | JN299302 |
| Sisyra nigra | FBNE001-11 | BC ZSM NEU 00001 | JN299301 |
| Sisyra terminalis | FBNE044-11 | BC ZSM NEU 00044 | JN299340 |
| Sisyra terminalis | FBNE043-11 | BC ZSM NEU 00043 |  |
| Sisyra terminalis | FBNE042-11 | BC ZSM NEU 00042 | JN299339 |
| Sisyra terminalis | FBNE041-11 | BC ZSM NEU 00041 | JN299338 |
| Sisyra terminalis | FBNE057-11 | BC ZSM NEU 00057 | JN299352 |
| Subilla confinis | FBNE113-13 | BC ZSM NEU 113 | KJ592476 |
| Sympherobius elegans | FBNE169-13 | BC ZSM NEU 169 | KJ592444 |
| Sympherobius fuscescens | FBNE101-13 | BC ZSM NEU 101 | KJ592557 |
| Sympherobius klapaleki | FBNE170-13 | BC ZSM NEU 170 |  |
| Sympherobius klapaleki | FBNE228-13 | BC ZSM NEU 228 | KJ592572 |
| Sympherobius pellucidus | FBNE229-13 | BC ZSM NEU 229 | KJ592474 |
| Sympherobius pellucidus | FBNE160-13 | BC ZSM NEU 160 | KJ592438 |
| Sympherobius pygmaeus | FBNE264-13 | BC ZSM NEU 264 | KJ592485 |
| Sympherobius pygmaeus | FBNE215-13 | BC ZSM NEU 215 | KJ592472 |
| Sympherobius pygmaeus | FBNE171-13 | BC ZSM NEU 171 | KJ592512 |
| Wesmaelius concinnus | FBNE099-13 | BC ZSM NEU 99 | KJ592507 |
| Wesmaelius concinnus | FBNE100-13 | BC ZSM NEU 100 | KJ592527 |
| Wesmaelius malladai | FBNE199-13 | BC ZSM NEU 199 | KJ592445 |
| Wesmaelius malladai | FBNE165-13 | BC ZSM NEU 165 | KJ592578 |
| Wesmaelius mortoni | FBNE159-13 | BC ZSM NEU 159 |  |
| Wesmaelius nervosus | FBNE094-11 | BC ZSM NEU 00094 |  |
| Wesmaelius nervosus | FBNE166-13 | BC ZSM NEU 166 | KJ592455 |
| Wesmaelius nervosus | FBNE200-13 | BC ZSM NEU 200 | KJ592576 |
| Wesmaelius quadrifasciatus | FBNE164-13 | BC ZSM NEU 164 | KJ592582 |
| Wesmaelius ravus | FBNE167-13 | BC ZSM NEU 167 |  |
| Wesmaelius subnebulosus | FBNE168-13 | BC ZSM NEU 168 | KJ592492 |
| Xanthostigma xanthostigma | FBNE238-13 | BC ZSM NEU 238 | KJ592580 |
| Xanthostigma xanthostigma | FBNE134-13 | BC ZSM NEU 134 |  |
